# Enhanced Viral Metagenomics with Lazypipe 2

**DOI:** 10.3390/v15020431

**Published:** 2023-02-04

**Authors:** Ilya Plyusnin, Olli Vapalahti, Tarja Sironen, Ravi Kant, Teemu Smura

**Affiliations:** 1 Department of Veterinary Biosciences, University of Helsinki, 00014 Helsinki, Finland; 2 Department of Virology, University of Helsinki, 00014 Helsinki, Finland; 3 HUS Diagnostic Center, Clinical Microbiology, Helsinki University Hospital, University of Helsinki, 00029 Helsinki, Finland; 4 Department of Tropical Parasitology, Institute of Maritime and Tropical Medicine, Medical University of Gdansk, 81-519 Gdynia, Poland

**Keywords:** metagenomics, virus, discovery, virome, mNGS, bioinformatics, pipeline

## Abstract

Viruses are the main agents causing emerging and re-emerging infectious diseases. It is therefore important to screen for and detect them and uncover the evolutionary processes that support their ability to jump species boundaries and establish themselves in new hosts. Metagenomic next-generation sequencing (mNGS) is a high-throughput, impartial technology that has enabled virologists to detect either known or novel, divergent viruses from clinical, animal, wildlife and environmental samples, with little a priori assumptions. mNGS is heavily dependent on bioinformatic analysis, with an emerging demand for integrated bioinformatic workflows. Here, we present Lazypipe 2, an updated mNGS pipeline with, as compared to Lazypipe1, significant improvements in code stability and transparency, with added functionality and support for new software components. We also present extensive benchmarking results, including evaluation of a novel canine simulated metagenome, precision and recall of virus detection at varying sequencing depth, and a low to extremely low proportion of viral genetic material. Additionally, we report accuracy of virus detection with two strategies: homology searches using nucleotide or amino acid sequences. We show that Lazypipe 2 with nucleotide-based annotation approaches near perfect detection for eukaryotic viruses and, in terms of accuracy, outperforms the compared pipelines. We also discuss the importance of homology searches with amino acid sequences for the detection of highly divergent novel viruses.

## 1. Introduction

Fast development in metagenomic Next-generation sequencing (mNGS) and analysis has enabled virologists to assess the true diversity of viruses in clinical, animal, wildlife and environmental samples. mNGS is a high-throughput, impartial technology with many advantages compared to established diagnostic methods for virus detection [1]. mNGS can detect viruses that do not propagate in cell cultures and, unlike PCR- or antigen-based detection, can detect a broad spectrum of viruses without a priori assumptions about the likely targets. Overall, mNGS has the potential to translate into a universal method for virus discovery, surveillance and broad-spectrum clinical diagnostics [1,2,3]. That said, we should note that mNGS is a relatively novel technology and there are still challenges to address such as accessibility, costs, and sampling to reporting time.

There is a growing interest within virology in utilizing mNGS, specifically in the detection of viruses that cannot be cultured [4,5]. There is also a growing interest for applications in clinical settings, particularly for difficult to diagnose cases with rare or unknown disease etiologies that would otherwise require multiple targeted tests [6,7]. mNGS is also recognized for its potential for the monitoring and early detection of emerging viral pathogens [1].

mNGS approaches are heavily dependent on bioinformatic analysis that processes raw sequence output by the NGS sequencer into metagenomic assemblies and into various reports on the micro-organisms’ nucleic acid presence and relative abundances in the analyzed samples. Generally, analysis of NGS data requires bioinformatic expertise, computational resources and, in many cases, installation and maintenance of large reference databases [2,3,7]. This has raised concerns that the lack of such in public health laboratories or smaller research facilities can cause hurdles against the adoption of mNGS methods [2,3,7]. These challenges can be addressed by developing bioinformatic pipelines and services designed to handle bioinformatic and resource-related challenges in mNGS sequence analysis. During the last decade many pipelines for virus discovery and sample composition analysis have emerged: VMGAP [8], PathSeq [9], VIROME [10], READSCAN [11], VirusFinder [12], SURPI [13], MetaVir [14], VIP [15], MetaShot [16], VirusSeeker [17], viGEN [18], Genome Detective [19], Kraken2 [20], IDseq [2] and Microseek [21]. mNGS pipelines tested in clinical settings are also beginning to emerge [7].

Our research group has contributed to this development with the Lazypipe mNGS pipeline designed primarily for virus discovery from clinical, animal and environmental samples with minimal requirements in terms of bioinformatic expertise and/or resources [22]. Lazypipe has been adopted as a standard module for mNGS analysis at the Finnish IT Center for Science (www.csc.fi, accessed on 21 December 2022) and has been successfully applied to detect and characterize a multitude of novel viral pathogens from a variety of different sample types, including arthropod vectors [23,24], mosquitos [25], farm animals [26] and wildlife [27]. Here, we present an enhanced version of our mNGS pipeline, Lazypipe 2. In this latest version, we introduce code updates to achieve better installation experience, stability, speed and transparency, as well as a smaller memory and disk-space footprint. We have also added new functionalities to support new analysis options and further automation of frequent user cases. New features include support for SPAdes assembler [28], minimap2 annotations [29], second round annotations with NCBI blastn [30], support for massively parallel execution for large sample batches and an alternative interface implemented with Snakemake [31]. Furthermore, we compiled a novel canine simulated metagenome and performed extensive benchmarking on human and canine simulated metagenomes. Using our benchmarks we also analyzed errors in virus detection and were able to detail sources of these errors, as well as compare nucleotide and amino acid based annotation strategies.

## 2. Materials and Methods

### 2.1. Code Updates

#### 2.1.1. Code Restructuring for Better Stability and Transparency

**Figure 1:**
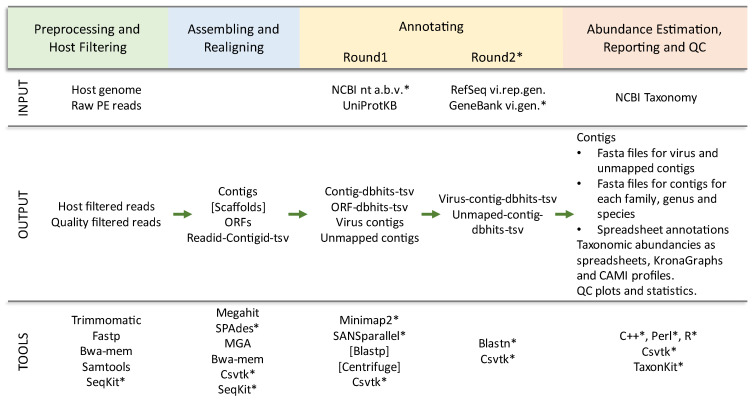
Overview of Lazypipe 2 workflow and updates. * Tools and databases that were added or updated in this release compared to the previously published work [22]. INPUT, input files and reference databases; dbhits, database homologs returned by the search; NCBI nt a.b.v., viral, bacterial and archaeal entries from the NCBI nt database; RefSeq vi.rep.gen., RefSeq representative virus genomes; GeneBank vi.gen., GeneBank complete virus genomes.

To improve the portability of Lazypipe1 (all versions freely available at https://bitbucket.org/plyusnin/lazypipe/, accessed on 21 December 2022), we excluded several external Perl modules that posed installation challenges to our users. Among other factors, we removed dependencies on spreadsheet modules and BioPerl modules. Spreadsheet modules were replaced with the R openxlsx library. All sequence manipulations with BioPerl were reimplemented with SeqKit calls [32]. All parsing and handling of taxonomic paths were reimplemented with TaxonKit calls [33]. To improve stability and transparency, all manipulations with tab-separated value (tsv) files were reimplemented using csvtk toolkit [34]. All three tools mentioned here (SeqKit, TaxonKit and csvtk toolkit) were selected based on similar criteria, namely simple installation without dependencies, speed and support for multithreading. These and other updates are illustrated in Figure 1.

Both Lazypipe 1 and 2 use SANSparallel [35], a fast, ~100 times faster than blastp, homology search for amino acid sequences (aa) against UniProtKB database. SANSparallel is set as the default search and is accessed using Pannzer [36] queries to the SANSparallel server. Integration of Lazypipe with SANSparallel was improved by adding a taxonomy operator to the Pannzer package (version 3.0). This operator handles mapping of UniProtKB accession ids to NCBI taxon ids on the SANSparallel server, which removed the requirement to perform this mapping locally by loading large accession to taxon id dictionaries into memory. This reduced the size of the code and also required memory.

The new version outputs several collections of contigs in fasta format: contigs mapped to viruses, contigs mapped to bacteriophages and contigs with no mapping (contigs_vi.fa, contigs_phages.fa and contigs_un.fa, respectively). The new version also outputs contig fasta files for each family, genus and species found in the data.

#### 2.1.2. Support for Parallel Analysis of Large Data Collections

We added support for massive parallel analysis of large sample batches using, for example, the Slurm job array framework. To address the analysis of large data-collections, all output fastq files are now compressed. We also added support for automated cleaning of intermediated files (activated with ‘clean option’). These later additions have significantly reduced the disk footprint of the pipeline.

### 2.2. New Features

#### 2.2.1. Integrating SPAdes

We added support for read assembling with SPAdes [28], which was shown in several comparative studies to have high performance for simulated and mock community viral metagenomes [37,38].

#### 2.2.2. Integrating Minimap2 Aligner

Lazypipe1 (version 1.0) supported annotation with both amino acid (aa) and nucleotide (nt) based search engines, using SANSparallel/blastp and Centrifuge, respectively. (SANSparallel searches with orfs against UniProtKB, blastp with orfs against local nr database and centrifuge with contigs against local nt database.) For the new version, we added support for the minimap2 nt search engine [29]. One practical consideration here was the constantly growing size of the NCBI nt database. For the June 2022 update, our attempt to run the Centrifuge indexer on the bacteria and virus portion of the nt database failed to reach completion after running for 70 h on 32 cores. Minimap2 was an attractive alternative, since this search engine supports database indexing in parts, thereby avoiding memory limitations. Minimap2 supports assembly to reference alignments (--x asm5/asm10/asm20 modes) with different expected sequence divergence. By default, Lazypipe 2 uses --xasm20 (approximately 5% divergence), and as our reference we use a custom database covering all viral, bacterial and archaeal entries from the NCBI nt database.

#### 2.2.3. Integrating Blastn for Second Round Annotations

The end users will often want to confirm virus annotations produced with fast aligners such as SANSparallel or minimap2 with a classical blastn search. To support this, we added the --pipe blastv option, which will run blastn on contigs identified as virus contigs in the main annotation round. As the reference database, the user can choose any custom or public blastn database. We recommend using RefSeq representative genomes for viruses, updated and published by NCBI [30]. We also offer support for a broader virus database that we compile and update from GeneBank complete virus genomes (https://bitbucket.org/plyusnin/lazypipe/, accessed on 21 December 2022). We also added support for re-annotation of contigs, which had no database hits in the main annotation round (contigs_un.fa). Unmapped contigs can be re-annotated with blastn against a custom database with --pipe blastu option.

#### 2.2.4. Improved Bacteriophage Labelling and Annotation

We added a more complete labelling of bacterial and archaeal viruses. The new labelling lists all viral families and orders, which include exclusively viral species known to infect bacteria or archaea according to the latest Virus Metadata Resource published by the International Committee on Taxonomy of Viruses (https://ictv.global/taxonomy/, accessed on 21 December 2022, VMR_20-190822). The labelling was made updatable and transparent by listing these bacteriophage families and orders in a separate source file (R/NGS.phage.filter.R).

We also added an option (--pipe annph) to run a 2nd round annotation of unmapped contigs (contigs_un.fa) with minimap2 against a local bacteriophage database.

#### 2.2.5. New Interface with Snakemake

To support a wider range of users, including those unfamiliar with perl, we complemented the default perl interface with an optional Snakemake interface [31]. Snakemake is a workflow manager that is able to handle large bioinformatic workflows with complex input–output interdependencies [31].

### 2.3. Benchmarking

We evaluated Lazypipe 2 on two benchmarks. For the first benchmark we used the human simulated metagenome from the MetaShot project [16]. This MetaShot metagenome is a simulated 20.5 M PE 2 × 150 Illumina library covering approximately 80 viral and 70 bacterial pathogens imbedded in a background of human reads [16]. For the second benchmark we compiled a novel canine simulated metagenome (described below), based on viruses and bacteria associated with the domestic dog. The domestic dog was chosen in order to test virus detection against a different host and bacterial background. The resulting simulated metagenome can also serve as a valuable tool for future mNGS benchmarking, particularly in the context of companion animal and one-health research.

All benchmarking was performed on a Linux/Unix CPU supercluster with 32 cores each running at 2.1 GHz.

#### 2.3.1. Human Simulated Metagenome

Human simulated metagenome was used to evaluate Lazypipe 2 (version 2.1) against Lazypipe1 (version 1.0), Kraken2 [20], CZID [2] and Genome Detective [19]. Lazypipe 2 was run with default options and minimap2 and SANSparallel aligners. Lazypipe1 was run with default options and SANSparallel aligner. Kraken2 was run with default settings and the standard database. CZID was run via web interface v7.1 with host set to “human” and background to “none”. Genome Detective Virus Tool was run via web interface v2.48 with default options and with host reads pre-filtered in order to pass the input size limit for the public interface.

#### 2.3.2. Viral Genomes Associated with the Domestic Dog

We started by searching RefSeq (version 214) for virus assemblies with host field matching to “Canis lupus”, “Canis lupus familiaris” or “dog”, or virus name matching “Canine”. This resulted in 44 accessions including 30 complete genomes and 14 complete cds sequences. We then complemented this list by searching VirusHostDB [39] for viruses that were labelled with “Canis lupus familiaris” as their host. From this list we manually selected assemblies for viruses that are either well established canine pathogens (e.g., Lyssavirus rabies) or that have been isolated from a dog. We further extended our collection by adding Canine Influenza A virus *H3N2* from the NCBI Influenza Virus Sequence Database. The H3N2 subtype is the latest and most common Influenza virus isolated from dogs in Asia and the United States [38]. Lastly, we pruned the list of collected canine papillomaviruses to include only one genome for each species-level taxon. This resulted in 7 canine papillomaviruses. The resulting collection of canine viral genomes included 57 assemblies and 39 unique virus taxa (accessions available in File S1).

#### 2.3.3. Bacterial Genomes Associated with the Domestic Dog

Bacterial genomes were added in order to make the canine simulated metagenome closer to real-world mNGS libraries, which almost invariably include bacterial sequences. Additionally, we were interested in benchmarking Lazypipe performance for the detection and annotation of bacteria, although the main focus remained on viral metagenomics.

We started by datamining bio-sample entries from the NCBI BioSample database that were labelled with host_taxid equal to 9615 or with host matching “(dog[s]?)|(canis lupus familiaris)”. This returned 21,083 unique dog-associated samples. We then selected from RefSeq genomes database (version 214) all bacterial accessions that were sequenced from dog-associated samples. We further pruned this list to include only unique taxon ids. This resulted in a collection of 195 accessions and 159 bacterial species. We also created a smaller collection that included only complete genomes from the above set (58 accession). Accessions and other details for both canine bacterial collections are available in File S1.

#### 2.3.4. Canine Simulated Metagenome

To create the canine simulated metagenome we processed our viral, bacterial and host (GCF_000002285.5) sequences with ART [40], applying settings for Illumina PE 2X 150 nt libraries with HiSeq 2500 built-in profile. Host genome and bacterial genomes were processed with 5X coverage, while viral genomes were processed with coverage ranging from 1X to 5X. We then combined host, bacterial and viral libraries into a number of canine metagenomes with varying proportions of viral and bacterial sequences and varying viral coverage (Table 1). Low proportion/coverage of virus genomes aimed to test virus calling at low to very low abundance of virus genetic material. Canine simulated metagenomes were then used to benchmark taxa calling and read taxonomic binning with Lazypipe 2 --ann minimap.

## 3. Results

### 3.1. Benchmarking Taxonomic Profiling

#### 3.1.1. Results for Human Simulated Metagenome

We compared Lazypipe 2 with minimap2 and SANSparallel aligners to Lazypipe1 with SANSparallel aligner, Kraken2, CZID and Genome Detective on the human simulated metagenome. Precision, recall and F1-score (harmonic mean of precision and recall) for predicted virus and bacterial taxa are given in Table 2 and Table 3, respectively. For viruses we excluded the least abundant taxa that accounted for the last percentile of read distribution. Similarly, for bacteria we excluded the least abundant taxa that accounted for the last five percentiles of the read distribution. As discussed previously [22], these settings aim to reduce noise for taxa at lower abundances. For bacterial predictions we also included Lazypipe 2 results pruned by the least abundant taxa that accounted for the last 20 percentiles of the read distribution (Lazypipe 2 --ann sans -t20) (Table 3). Pruning of the least abundant taxa was done for all tools, except the Genome Detective, and had a positive effect on accuracy.

Lazypipe 2 with minimap2 aligner demonstrated the best accuracy for virus calling with recall at 95.2% and precision at 97.5% (species-level, Table 2). Lazypipe 2 with SANSparallel aligner was the fourth best with recall at 89.2% and precision at 85.1% (Table 2).

Lazypipe 2 with SANSparallel (Lazypipe 2 --ann sans -t20) had the overall best accuracy for bacterial calling (90.1% recall and 84.2% precision). Notably, the high precision was achieved by pruning the last 20 percentiles from the result list. This had a large effect by significantly decreasing the false positive bacterial predictions compared to the default pruning of 5 percentiles (Lazypipe --ann sans with 39.1% precision). Lazypipe 2 with minimap2 aligner was second best (64.8% recall and 61.3% precision) followed by Lazypipe1 --ann sans, IDseq, Lazypipe 2 --ann sans and Kraken2.

#### 3.1.2. Classification Errors for Human Simulated Metagenome

To gain a better understanding of errors in virus detection, these were examined in more detail. We focused on erroneous calls for eukaryotic viruses reported by Lazypipe 2 with SANSparallel and minimap2 aligners (Table 4). These represent annotations by sequence homology of nucleotide (nt) contig sequences (minimap2) and amino acid (aa) sequences in open reading frames (SANSparallel). We also refer to these as the nt- and the aa-based annotations, respectively.

Erroneous calls were due to a limited number of typical causes (Table 4). Detailed descriptions of all misclassification errors are available in File S2. Here we report the key points.

For endogenous retroviruses, false-negatives were caused by host-genome filtering which, in most cases, also removed the retroviral reads. These errors can be avoided by turning host-filtering off.

For the aa-based annotation, common false positive errors were due to homologs in the aa space (labelled as “orf with high identity to FP” in Table 4) and common false negative errors were due to the failure to predict correct orfs (labelled as “orf prediction” in Table 4). Naturally, these errors did not occur in the nt-based annotations.

There were also cases of contigs having similar identity to both true and false positives (labelled as “Contig with high identity to FP”). For example *Naples phlebovirus* (false negative) and *Toscana Phlebovirus* (false positive) were identical within the assembled region.

There was a single case of mis-assembling two closely related viruses (*Mopeia Lassa virus reassortant 29* and *Lassa virus*) as a single contig leading to a false negative error.

#### 3.1.3. Results for Canine Simulated Metagenome

We benchmarked our pipeline on canine simulated metagenome with default options and minimap2 annotation. Pipeline demonstrated 100% recall of virus species for all variants of the canine metagenome except the lowest 1X coverage version, for which there was a single false negative (Table 5). Pipeline called a single false positive virus prediction for all metagenome versions. For the dog5X-ba5X-vi1-5X series, false positive was the uncultured human fecal virus (UHFV, taxid 239364). UHFV was called with 15 contigs, which all originated from *Bifidobacterium pseudocatenulatum* assembly (GCF_022496265.1). We hypothesize that this may represent an unclassified bacteriophage genome. For the dog5X-ba.comp5X-vi5X metagenome false positive was for the *Human gammaherpesvirus 4* (syn. *Epstein-Barr virus*, EBV) with just 18 reads. This originated from *Corynebacterium amycolatum* assembly (NZ_CP102778.1), which was added to RefSeq at a later time point (23 August 2022) than the compilation of our reference database (20 June 2022). There were fewer errors in virus recall from the canine metagenome compared to human metagenome, although this difference was minor. Additional errors for the human metagenome were due to included retroviruses, misclassification of two closely related phlebo-viruses and mis-assembly of *Arenavirus* and *Mammarenavirus* reads into the same contig.

Similar to benchmarking on the human simulated metagenome, retrieval of bacterial taxa was evaluated, ignoring the least abundant taxa that accounted for the last 5 percentiles of read distribution. The accuracy for bacteria was comparable to human metagenome results. Recall and precision for bacterial genera were relatively high: 85.9–96.9% and 95.3–96.9%, respectively (Table 5).

### 3.2. Benchmarking Read Binning and Genome Coverage

We evaluated read binning and genome coverage from Lazypipe 2 --ann minimap results for the canine simulated metagenome (dog5X-ba5X-vi5X). For each viral genome we compared read ids of the simulated reads and reads assigned by the pipeline. From these numbers, we estimated recall and precision for read taxonomic binning. For 64% of viral genomes, both recall and precision for read binning exceeded 99%, and for 85% of viral genomes these exceeded 80% (Table S1).

**Figure 2:**
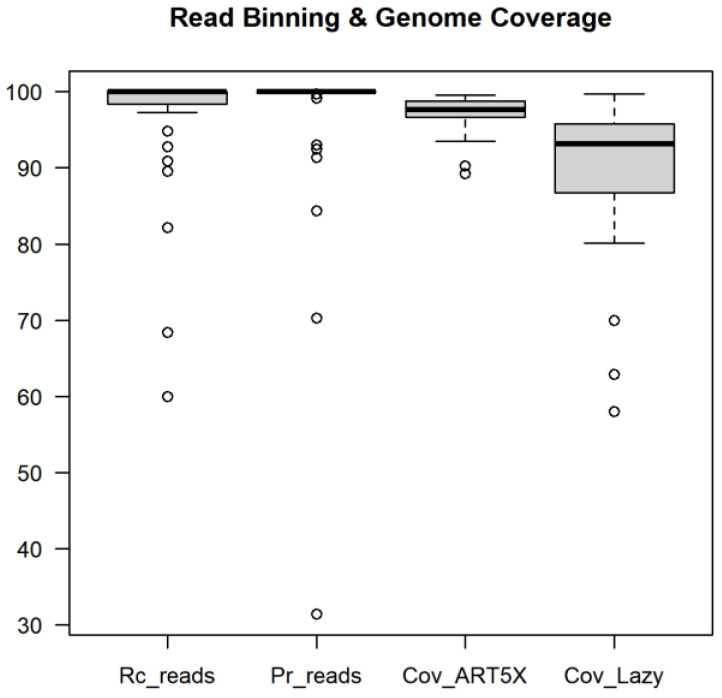
Benchmarking read binning and genome coverage. The first two boxplots represent recall and precision of read taxonomic binning for viral genomes. The last two columns represent genome coverage by the simulated reads (Cov_ART5X) and by the Lazypipe assemblies (Cov_Lazy).

We also estimated horizontal coverage of viral genomes by the resulting assemblies. The pipeline created assemblies for 37 out of 39 unique virus taxids in the benchmark. The exceptions were the three canine parvovirus genomes that were all assembled to a single genome. For these 37 genomes, the median coverage by the simulated reads and Lazypipe assemblies was 97.7% (96.7–98.8%) and 92.3% (86.7–95.8%), respectively (Figure 2).

### 3.3. Benchmarking Time Performance and Disk Footprint

We compared the execution time of Lazypipe 2 (with options --ann minimap and --ann sans) to Lazypipe1 (with option --ann sans), Kraken2 and Genome Detective. Benchmarking was carried out with human simulated metagenome [16] on Linux/Unix CPU supercluster with 32 cores each running at 2.1 GHz. Genome Detective Virus Tool was run via web interface v2.48 with default options and with host-reads pre-filtered. Real and CPU times for the compared tools are displayed in Table 6. The new version of the pipeline was slightly slower than v1.0. This is mainly due to overhead introduced by fastq file compression. The main bottleneck was the database search. For both Lazypipe1 --ann sans and Lazypipe 2 --ann sans, database search accounted for 70–71% of real execution time.

The reduction of disk footprint for the human simulated metagenome analysis with Lazypipe 2 versus Lazypipe1 was approximately two-fold.

## 4. Discussion

Metagenomic analysis pipelines are vital for global pathogen detection and monitoring. Here, we presented Lazypipe 2, an updated version of our mNGS pipeline with significant improvements in code ability, transparency and support for new software components. The previous version, Lazypipe, and now Lazypipe 2 have been used, contributing to virus discovery and demonstrating its potential for unbiased NGS-based studies [23,24,25,26,27].

Benchmarking on simulated metagenomes demonstrated that assembling and taxonomic binning of contigs with minimap2 against a subset of NCBI nt is a highly accurate strategy for calling known viruses. For the human simulated metagenome Lazypipe 2 --ann minimap achieved 95.2% recall and 97.5% precision for viral species. For the canine simulated metagenome Lazypipe 2, --ann minimap had 100% recall and 97.2% precision. Notably, this high accuracy was sustained even with a heavy host and bacterial sequence background with viral reads spiked at just 1X coverage and constituting just 0.004% of the total NGS library. Additionally, we demonstrated that viral genomes assembled from viral reads spiked at 5X had good horizontal coverage (median at 92%). In addiction, recovery of the spiked reads for viral genomes was highly accurate (precision and recall exceeding 99%) for two thirds of viral genomes and at high level (>80%) for 85% of viral genomes spiked at 5X.

Annotating with SANSparallel (a homology search for aa sequences) had a slightly lower performance for calling known viruses from the human simulated metagenome. On this benchmark, Lazypipe 2 --ann sans showed 89.2% recall and 85.1% precision for viral species. For annotations based on aa sequences, most errors were failures to identify measurable orfs and misalignments of orfs to false positives. For nt-based annotations, misalignment to false positives were limited to just two cases, one due to mis-assembly and one due to identical genomic regions in closely related viruses. These observations support the choice of nt-based annotations for known viral targets with low divergence from reference sequences. Possible scenarios for applying nt-based annotations include surveillance of a list of known pathogens from various samples and diagnostics or research targeting known viruses with clinical samples.

Annotations based on aa sequences have higher sensitivity for viruses with higher divergence from the reference [2,17,21]. We must also consider that current reference databases are estimated to represent only a fraction of viral diversity [41]. These points advocate in favor of homology search with aa sequences when looking for novel and divergent viruses. However, there is a trade-off between finding potential new viruses with relatively low aa identity and misclassification of host, environmental or bacterial sequences as potential viruses.

Identification of known viruses with Lazypipe 2 --ann minimap approached perfect accuracy in detecting viruses from simulated metagenomes. The remaining errors were caused by filtering of endogenous retroviruses with the host reads and close homologs that were identical within the assembled region. Correct identification of endogenous retroviruses prior to host filtering is an important goal for future development. This study also left out benchmarking on real datasets, although we expect the performance to be at least at the level of Lazypipe1 (tested on a mock-virome dataset).

Another important goal for future development is improving the detection of divergent novel viruses. This can be approached in several ways, for example, by integrating well established and highly sensitive techniques based on Hidden Markov Models for detecting protein homologs (e.g., HMMER [42]). Similar ideas have been implemented in other mNGS pipelines [21]. Higher sensitivity will pose challenges such as higher number of false positives and the nontrivial task of evaluating performance. Benchmarking the detection of novel divergent viruses is difficult to formulate, although some efforts have been made using methods for simulated evolution [2,21].

Most of the currently existing mNGS pipelines only support short-read sequencing platforms but we are planning to work further on Lazypipe 2 to support long-read platforms (e.g., Oxford Nanopore Technologies, which is becoming highly popular for pathogen surveillance due to its portability and cost effectiveness) and make it more user friendly by developing a web interface for fairly self-explanatory results in Hypertext Markup Language. This will help scientists and clinicians with minimum bioinformatics skills to analyze their samples and gain insight from mNGS datasets for both known and novel pathogens.

## Figures and Tables

**Table 1:** Canine simulated metagenomes. We combined the host library, two bacterial libraries and viral libraries at different coverage levels (5X to 1X).

Metagenome	Host Reads	Bacterial Reads	Virus Reads	Bacterial Taxids	Virus Taxids
dog5X-ba.comp5X-vi5X	93.37%	6.60%	0.021%	58	39
dog5X-ba5X-vi5X	79.85%	20.13%	0.018%	195	39
dog5X-ba5X-vi4X	79.85%	20.13%	0.014%	195	39
dog5X-ba5X-vi3X	79.86%	20.13%	0.011%	195	39
dog5X-ba5X-vi2X	79.86%	20.13%	0.007%	195	39
dog5X-ba5X-vi1X	79.86%	20.14%	0.004%	195	39

**Table 2:** Accessing accuracy of virus taxon retrieval by different tools. Compared tools are ordered by the descending F1-score. * Evaluation of Genome Detective excluded three Human endogenous retroviruses that may have been pre-filtered with host-reads. True, number of virus taxa in the benchmark, TP, number of true positive predictions, FP, number of false positive predictions, FN, number of false negative predictions, Pr, precision, Rc, recall, F1, F1-score.

Tool	Rank	True	TP	FP	FN	Pr	Rc	F1
Lazypipe 2 --ann minimap	species	83	79	2	4	97.5%	95.2%	96.3%
IDseq	species	83	77	4	6	95.1%	92.8%	93.9%
GenomeDetectiveVirus *	species	80	75	5	5	93.8%	93.8%	93.8%
Lazypipe 2 --ann sans	species	83	74	13	9	85.1%	89.2%	87.1%
Lazypipe1 --ann sans	species	83	73	13	10	84.9%	88.0%	86.4%
Kraken2	species	83	75	18	8	80.6%	90.4%	85.2%
Lazypipe 2 --ann minimap	genus	48	47	0	1	100.0%	97.9%	98.9%
IDseq	genus	48	46	0	2	100.0%	95.8%	97.9%
GenomeDetectiveVirus *	genus	48	46	1	2	97.9%	95.8%	96.8%
Kraken2	genus	48	46	1	2	97.9%	95.8%	96.8%
Lazypipe 2 --ann sans	genus	48	44	1	4	97.8%	91.7%	94.6%
Lazypipe1 --ann sans	genus	48	42	1	6	97.7%	87.5%	92.3%

**Table 3:** Accessing accuracy of bacterial taxon retrieval by different tools. Compared tools are ordered by the descending F1-score. True, number of bacterial taxa in the benchmark, TP, number of true positive predictions, FP, number of false positive predictions, FN, number of false negative predictions, Pr, precision, Rc, recall, F1, F1-score.

Tool	Rank	True	TP	FP	FN	Pr	Rc	F1
Lazypipe 2 --ann sans -t20	species	71	64	12	7	84.2%	90.1%	87.1%
Lazypipe 2 --ann minimap	species	71	46	29	25	61.3%	64.8%	63.0%
Lazypipe1 --ann sans	species	71	68	79	3	46.3%	95.8%	62.4%
IDseq	species	71	42	24	29	63.6%	59.2%	61.3%
Lazypipe 2 --ann sans	species	71	70	109	1	39.1%	98.6%	56.0%
Kraken2	species	71	37	62	34	37.4%	52.1%	43.5%
Lazypipe 2 --ann sans -t20	genus	44	41	4	3	91..1%	93.2%	92.1%
Lazypipe1 --ann sans	genus	44	44	9	0	83.0%	100.0%	90.7%
Lazypipe 2 --ann minimap	genus	44	34	5	10	87.2%	77.3%	81.9%
IDseq	genus	44	33	3	11	91.7%	75.0%	82.5%
Lazypipe 2 --ann sans	genus	44	44	18	0	71.0%	100.0%	83.0%
Kraken2	genus	44	29	9	15	76.3%	65.9%	70.7%

**Table 4:** Errors in virus detection from the human simulated metagenome. The table summarizes false-positive (FP) and false-negative (FN) errors (highlighted) reported by Lazypipe 2 with sans and minimap2 annotation options. Error causes, represented by the fourth column, are explained in the text. The last column represents the part of the pipeline where the error occurred.

Virus	Lazypipe --ann sans	Lazypipe --ann minimap2	Error Cause	Pipeline Step
*Human endogenous retrovirus*	FN	FN	Reads filtered as host reads	Preprocessing
*Human endogenous retrovirus W*	FN	FN		Preprocessing
*Naples phlebovirus*	FN	FN	Contig (4187 nt) with high (100%) identity to *Toscana phlebovirus*	DB search
*Mopeia Lassa virus reassortant 29*	FN	FN	Mis-assembled contig (1551 nt) with high (99.9%) identity to *Lassa virus*	Assembling
*Hepatitis B virus*	FN	TP	orf prediction	orf prediction
*Uukuniemi uukuvirus*	FN	TP	orf prediction	orf prediction
*Influenza A virus*	FN	TP	orf prediction	orf prediction
*Mason-Pfizer monkey virus*	FN	TP	Low read count	Reporting
*Toscana phlebovirus*	FP	FP	Contig (4187 nt) with high (100%) identity to FP	DB search
*Cowpox virus*	FP	TN	orfs with high (100%) identity to FP	DB search
*Borna disease virus*	FP	TN	orfs (429 nt, 606 nt and 1113 nt) with high (100%) identity to FP	DB search
*Central chimpanzee simian foamy virus*	FP	TN	orfs (668 nt and 696 nt) with high (96.0–99.0%) identity to FP	DB search
*Eastern chimpanzee simian foamy virus*	FP	TN	orfs (180–759 nt) with high (98.0–100%) identity to FP	DB search
*African green monkey simian foamy virus*	FP	TN	orf (753 nt) with high (81.1%) identity to FP	DB search
*Human bocavirus*	FP	TN	orfs (441–2016 nt) with high (100%) identity to FP	DB search
*Chimeric Tick-borne encephalitis virus/* *Dengue virus 4*	FP	TN	orf (6108 nt) with high (100%) identity to FP	DB search
*Phlebovirus SDYY104/China/2011 virus*	FP	TN	orf (6258 nt) with high (100%) identity to FP	DB search
*SFTS phlebovirus*	FP	TN	orf (738 nt) with high (100%) identity to FP	DB search
*Bocaparvovirus sp.*	FP	TN	orf (1920 nt) with high (100%) identity to FP	DB search
*Coronavirus BtRs-BetaCoV/YN2018D*	FP	TN	orf (966 nt) with high (100%) identity to FP	DB search
Uncultured human fecal virus	TN	FP	12 contigs (330–586 nt) with high identity to FP	DB search

**Table 5:** Accessing Lazypipe 2 --ann minimap accuracy on canine simulated metagenome. Here ba5X and ba.comp5X represent the canine bacterial genomes and canine complete bacterial genomes, respectively. True, number of ground truth taxa, TP, true positives, FP, false positives, FN, false negatives, Pr, precision, Rc, recall, F1, F1-metric.

Metagenome	Target Taxa	TRUE	TP	FP	FN	Pr	Rc	F1
dog5X-ba.comp5X-vi5X	Virus species	35	35	1	0	97.2%	100.0%	98.6%
dog5X-ba5X-vi5X	Virus species	35	35	1	0	97.2%	100.0%	98.6%
dog5X-ba5X-vi4X	Virus species	35	35	1	0	97.2%	100.0%	98.6%
dog5X-ba5X-vi3X	Virus species	35	35	1	0	97.2%	100.0%	98.6%
dog5X-ba5X-vi2X	Virus species	35	35	1	0	97.2%	100.0%	98.6%
dog5X-ba5X-vi1X	Virus species	35	34	1	1	97.1%	97.1%	97.1%
dog5X-ba.comp5X-vi5X	Bacterial species	53	40	9	13	81.6%	75.5%	78.4%
dog5X-ba.comp5X-vi5X	Bacterial genera	32	31	1	1	96.9%	96.9%	96.9%
dog5X-ba5X-vi5X	Bacterial species	159	96	82	63	53.9%	60.4%	57.0%
dog5X-ba5X-vi5X	Bacterial genera	71	61	3	10	95.3%	85.9%	90.4%

**Table 6:** Benchmarking time performance. Real and CPU execution times for different tools with the human simulated metagenome. * For Genome Detective host reads were pre-filtered. NA: not applicable.

Tool	Real	CPU
Kraken2	0:02:38	0:09:13
Lazypipe1 --ann sans	1:18:02	6:43:42
Lazypipe 2 --ann sans	1:35:34	6:45:42
Lazypipe 2 --ann minimap	1:47:21	10:37:09
Genome Detective *	2:01:49	NA

## Data Availability

Lazypipe 2 user manual, reference databases and source code are available at https://bitbucket.org/plyusnin/lazypipe/ (accessed on 21 December 2022) and https://www.helsinki.fi/en/projects/lazypipe (accessed on 21 December 2022). Canine simulated metagenome presented in this study is available at Zenodo (https://doi.org/10.5281/zenodo.7471995, (accessed on 21 December 2022), Creative Commons Attribution 4.0 International License).

## Supplementary Materials

The following supporting information can be downloaded at: https://www.mdpi.com/article/10.3390/v15020431/s1, File S1: Accession ids and other information for virus and bacterial genomes included in the canine simulated metagenome; File S2: Misclassification errors by Lazypipe 2 --ann minimap and --ann sans for human simulated metagenome; Table S1: Results for viral read binning and genome coverage for canine simulated metagenome.
