# Supplementary material for: Enhanced Viral Metagenomics with Lazypipe 2
Source: Viruses. 2023 Feb 4;15(2):431. doi: 10.3390/v15020431 (PMC9960287; doi:10.3390/v15020431)
Supplement: Supplementary file 1 [file viruses-15-00431-s001.zip › Supp.v7.corrected/File-S2_class-errors.pdf]

File S2 for Enhanced Viral Metagenomics with Lazypipe2

Classification errors for human simulated metagenome

Human endogenous retrovirus and *Human endogenous retrovirus W* were not predicted. All reads from these genomes were filtered as host reads, since these aligned to the human genome.

Naples phlebovirus was misclassified as *Toscana phlebovirus* (false positive). Lazypipe2 assembled all 280 reads labelled in the benchmark as *N. phlebovirus* (NC_006320.1) to a single 4,187 nt contig (*k141.9952*) and identified a single 960 nt orf. Lazypipe2 mapped this orf to *Toscana phlebovirus* with 99.3% coverage and 99.0% identity. Mapping with blastn confirmed 100% contig identity to both *Naples* and *Toscana phlebovirus* M segment (acc NC_006320.1 and X89628.1). In this case, the assembled region was identical in the two closely related viruses.

Mopeia Lassa virus reassortant 29 was misclassified as *Lassa virus* (true positive). Lazypipe2 mis-assembled 220 reads from the *Mopeia Lassa virus reassortant 29* and 220 reads from *Lassa virus* (syn. *Lassa mammarenavirus*) to a single 3,375 nt contig (*k141.54025*) and identified a single 1,551 nt orf. Lazypipe2 mapped this orf to *Lassa virus* (top 3 hits) with 99.6% coverage and 100% identity. Mapping with blastn confirmed 99.94% contig identity to *Lassa virus* and 99.88% identity to *Mopeia Lassa virus reassortant 29*. In this case, the error was due to mis-assembling to a single contig and, as a result, false negative prediction for one of the viruses.

Hepatitis B virus was a false negative missing from the result list. Lazypipe2 assembled all 210 benchmark reads to a single 3,173 nt contig (*k141.36746*) and identified a short 317 nt orf. Lazypipe2 erroneously mapped this orf to *Trichinella spiralis.* Mapping with blastn confirmed 100% contig identity to *Hepatitis B virus* with 98.7% coverage of the target genome. In this case, the error was due to the gene-finding software that failed to identify correct ORFs.

Uukuniemi uukuvirus was a false negative missing from the result list. Lazypipe2 assembled all 210 benchmark reads to a single 3,199 nt contig (*k141.29920*) and identified two short orfs (132 nt and 279 nt). There were no significant database hits for these orfs. Mapping with blastn confirmed 100% contig identity to *Uukuniemi virus* with 99.1% coverage of the M-segment (acc M17417.1). Also, in this case gene-finding software failed to report expected orfs.

Influenza A virus was a false negative missing from the result list. Lazypipe2 assembled all 90 benchmark reads to a single 1,386 nt contig (*k141.46069*) and identified a single 105 nt orf. There were no significant database hits for this orf. Mapping with blastn confirmed 100% identity to *Influenza A virus* with 97.7% coverage of the neuraminidase gene (acc AJ404629.1). Also, in this case gene-finding software failed to report expected orfs.

Mason-Pfizer monkey virus (syn. *Human type D retrovirus*) was misclassified as *Squirrel monkey retrovirus* (false positive). Lazypipe2 assembled all 65 benchmark reads to a single 308 nt contig (*k141.29825*) and identified a single 306 nt orf. Pipeline mapped this orf to *Squirrel monkey retrovirus* (also a *Betaretrovirus*). However, this prediction was filtered from abundance estimation due to low read count. Mapping with blastn confirmed 100% identity to *Human type D retrovirus* (acc D10443.1).

Part of *Vaccinia virus* reads were erroneously assigned to *Cowpox virus* (false positive). Lazypipe2 reported *Cowpox* with 551 PE reads, a single 174,612 nt contig (*k141.74634*) and 157 orfs. Lazypipe2 mapped these orfs to various *Orthopox* viruses including *Camelpox virus*, *Cowpox virus, Monkeypox virus*, and *Vaccinia virus*. However, only *Cowpox* and *Vaccinia virus* were picked by the weighting model. Mapping with blastn confirmed 100% contig identity to *Vaccinia virus* (acc AY243312.1).

There was confusion with labelling *Borna disease viruses* (BDV). According to the original publication [1], the benchmark included 590 PE reads from *Borna disease virus* (taxid 12455). However, the corresponding reads were marked with *NC_001607* accession, which corresponds to *Borna disease virus 1* (taxid 1714621, considered here as the ground truth). Lazypipe2 assembled 589 PE reads labelled with NC_001607 accession to a 8,890 nt contig (*k141.57245*) and identified three orfs. Lazypipe2 mapped three orfs to *Borna disease virus* (taxid 12455, 98.6-99.5% query coverage and 100% identity) and two orfs to *Borna disease virus 1* (taxid

1714621, 98.6-99.5% query coverage and 100% identity) and reported both viruses. Mapping with blastn confirmed 100% contig identity to *Borna disease virus 1* (taxid 1714621).

Part of *Simian foamy virus (SFV)* reads were misclassified to *Central cimpanzee SFV* (false positive). Lazypipe2 reported this with 193 PE reads, one contig (*k141.72284*) and two orfs. The pipeline mapped these orfs to *SFV* (99.6% coverage and 99.0-100% identity) and *Central cimpanzee SFV* (99.1-99.6% coverage and 96.0-99.0% identity). Mapping with blastn confirmed 99.00-99.15% contig identity to *Human spumaretrovirus*, 98.50% identity to *Central cimpanzee SFV* (false positive) and 96.71%-98.21% identity to *SFV* (ground truth).

Part of *SVF* reads were also misclassified to *African green monkey SVF* (false positive). Lazypipe2 reported this with 32 PE reads, one contig and one 753 nt orf. This orf was mapped to *African green monkey SVF* with 97.2% coverage and 81.0% identity.

Another false positive was the *Eastern chimpanzee SFV.* Lazypipe2 reported this with 146 PE reads, three contigs (*k141.47714*, *k141.38283* and *k141.9270*) and four orfs. Mapping with blastn showed 99%-100% contig identity to *Human spumavirus* (not in the benchmark), 98.41-99.61% identity to the *Eastern chimpanzee SFV* and 85.03-96.75% identity to *SFV*.

Part of *Primate bocavirus 1* reads were misclassified by Lazypipe2 to *Human bocavirus* (false positive) and *Bocaparvovirus sp.* (false positive, taxid 1883111). Pipeline reported these with 154 and 36 PE reads, one 5,252 nt contig (*k141.64308*) and one to three orfs. Pipeline mapped three orfs to *Primate bocavirus 1*, three orfs (1,920 nt, 441 nt and 2,016 nt) to *Human bocavirus* (with 100% identity) and one orf (1,920 nt) to *Bocaparvovirus sp*. (100% identity). Mapping with blastn confirmed 100% contig coverage and identity to the ground truth *Primate bocaparvovirus 1*, 99.94% identity to *Human bocavirus* (false positive) and 99.90% identity to *Bocaparvovirus sp*. (false positive).

Lazypipe2 reported *Chimeric Tick-borne encephalitis virus/Dengue virus* 4 (false positive, taxid 638787) with 349 PE reads, one 10,624 nt contig (*k141.780*) and a single 6,108 nt orf. Pipeline mapped this orf to *Dengue virus 4* and the chimeric virus with identical scores, 99.9% query coverage and 100 % identity. Mapping with blastn showed that the contig was 100% identical to a fragment of *Dengue virus 4* genome. The error here is due to the orf beeing identical between the two viruses.

Lazypipe2 reported *Phlebovirus SDYY104/China/2011* virus (false positive, taxid 1848960) with 140 PE reads, one 6,353 nt contig (*k141.30190*) and a single 6,258 nt orf. Pipeline mapped this orf to *Phlebovirus SDYY104/China/2011* with 99.9% coverage and 100% identity. Mapping with blastn confirmed 100% identity of the contig to *Dabie bandavirus*.

Lazypipe2 misclassified some of *Dabie bandavirus* reads to *SFTS phlebovirus* (false positive, taxid 1933190). The pipeline reported this with 88 PE reads, a single 1,700 nt contig (*h*), and a single 738 nt orf. Pipeline mapped this orf to *SFTS phlebovirus* with 99.2% coverage and 100% identity.

References

1. Fosso, B.; Santamaria, M.; D'Antonio, M.; Lovero, D.; Corrado, G.; Vizza, E.; Passaro, N.; Garbuglia, A.; Capobianchi, M.; Crescenzi, M.; et al. MetaShot: An Accurate Workflow for Taxon Classification of Host-Associated Microbiome from Shotgun Metagenomic Data. *Bioinformatics* **2017**, *33*, 1730–1732.
