# Supplementary material for: Enhanced Viral Metagenomics with Lazypipe 2
Source: Viruses. 2023 Feb 4;15(2):431. doi: 10.3390/v15020431 (PMC9960287; doi:10.3390/v15020431)
Supplement: Supplementary file 1 [file viruses-15-00431-s001.zip › Supp.v7.corrected/Table-S1_benchmarking.pdf]

Virus	Taxid	Spike dcov	Spike readn	Rc	Pr	GenCov ART	GenCov Lazypipe2
Astrovirus dogfaeces/Italy/2005	566308	5X	45	82.2%	92.5%	98.2%	80.1%
Betapolyomavirus canis	1980633	5X	83	100.0%	100.0%	96.0%	89.4%
Canid alphaherpesvirus 1 (CHV)	170325	5X	2085	99.9%	100.0%	98.7%	86.7%
Canine adenovirus 1	10512	5X	1016	99.9%	99.7%	98.3%	99.7%
Canine adenovirus 2	10514	5X	520	99.4%	100.0%	99.4%	97.0%
Canine astrovirus	1157338	5X	110	97.3%	93.0%	98.5%	89.1%
Canine bocavirus 1	1511885	5X	90	100.0%	100.0%	97.1%	93.7%
Canine bocavirus 3	1295080	5X	88	100.0%	100.0%	97.0%	93.7%
Canine circovirus	1194757	5X	33	90.9%	100.0%	97.2%	70.0%
Canine kobuvirus	1836608	5X	135	89.6%	70.3%	95.9%	82.9%
Canine kobuvirus CH-1	1281440	5X	135	60.0%	84.4%	99.1%	58.0%
Canine kobuvirus US-PC0082	1082189	5X	138	92.8%	91.4%	99.2%	84.4%
Canine minute virus	329639	5X	83	100.0%	100.0%	99.3%	95.9%
Canine morbillivirus	11232	5X	260	100.0%	100.0%	99.2%	96.9%
Canine parvovirus	10788	5X	88	0.0%	0.0%	99.4%	0.0%
Canine parvovirus 2a	497961	5X	83	100.0%	31.4%	93.5%	95.8%
Canine parvovirus 2b	568986	5X	83	0.0%	0.0%	95.8%	0.0%
Canine picodicistrovirus	1150861	5X	145	100.0%	100.0%	97.7%	95.9%
Canine picornavirus	1196647	5X	130	100.0%	100.0%	98.2%	92.3%
Canine respiratory coronavirus	215681	5X	515	94.8%	100.0%	98.8%	87.7%
Canine vesivirus	1176484	5X	140	97.9%	100.0%	95.9%	85.6%
Canis familiaris papillomavirus 13	1226723	5X	135	98.5%	100.0%	96.2%	89.2%
Canis familiaris papillomavirus 2	2759772	5X	135	100.0%	100.0%	96.7%	93.5%
Canis familiaris papillomavirus 3	360397	5X	130	100.0%	99.2%	98.3%	94.6%
Canis familiaris papillomavirus 4	464980	5X	128	100.0%	100.0%	97.4%	94.3%
Canis familiaris papillomavirus 6	1513269	5X	135	100.0%	100.0%	99.3%	96.3%
Canis familiaris papillomavirus 8	1081055	5X	128	98.4%	100.0%	97.0%	90.0%
Deltapolyomavirus canis	2170403	5X	85	100.0%	100.0%	97.5%	96.3%
Faeces associated gemycircularvirus 1	1843735	5X	35	100.0%	100.0%	89.3%	89.3%
Influenza A virus (H3N2)	11320	5X	213	100.0%	100.0%	94.6%	88.4%
Lambdapapillomavirus 2	35258	5X	143	99.3%	100.0%	99.6%	94.6%
Lupine bocavirus	2017714	5X	85	100.0%	100.0%	98.6%	97.1%
Lyssavirus rabies	11292	5X	198	99.5%	100.0%	98.1%	93.2%
Norovirus dog/GVI.1/HKU_Ca026F/20	673457	5X	125	100.0%	100.0%	98.9%	94.6%
Parainfluenza virus 5	2905673	5X	253	68.4%	100.0%	97.3%	62.9%
Picobirnavirus dog/KNA/2015	1961162	5X	28	100.0%	100.0%	90.3%	86.0%
Pneumovirus dog/Bari/100-12/ITA/20	2482952	5X	248	100.0%	100.0%	99.0%	97.6%
Rotavirus I	1637496	5X	288	99.7%	100.0%	93.9%	86.1%
Torque teno canis virus	687385	5X	45	100.0%	100.0%	97.0%	93.8%

Table S1. Benchmarking read binning and genome coverage. Viral genomes reported by Lazypipe2 –*ann minimap* for the canine simulated metagenome. Spike dcov, depth of genome coverage set in ARK simulation, Spike readn, number of reads output by ARC simulation, Rc, recall, Pr, precision, GenCov ART, genome coverage by simulated reads, GenCov Lazypipe2, genome coverage by Lazypipe2 assemblies.
